# FUNCTIONAL LIMITATIONS AND ABSENCE OF CARE AMONG OLDER ADULTS LIVING WITH DEMENTIA IN CHINA, ENGLAND, THE EUROPEAN UNION, AND THE UNITED STATES

**DOI:** 10.1093/geroni/igad104.0168

**Published:** 2023-12-21

**Authors:** Zhuoer Lin, Yuting Qian, Thomas Gill, Xiaohui Hou, Shanquan Chen, Xi Chen

**Affiliations:** Yale University, New Haven, Connecticut, United States; Yale University, New Haven, Connecticut, United States; Yale School of Medicine, New Haven, Connecticut, United States; The World Bank, Washington, District of Columbia, United States; London School of Hygiene & Tropical Medicine, London, England, United Kingdom; Yale University, New Haven, Connecticut, United States

## Abstract

Assistance with daily living is essential for people living with dementia (PLWD) who also have functional limitations. Despite the growing demands for caregiving, the absence of care for PLWD with functional limitations can be large and concerning. Using harmonized longitudinal survey data (2012-2018) on community-dwelling adults aged 50+ from the US, England, Europe (covering 28 countries), and China, we provided the first comparative evidence on the global trends in functional limitations and absence of caregiving for PLWD. We demonstrated three alarming facts in both developing and developed countries: there was an overall increasing trend in the extent of functional limitations defined as difficulty with basic or instrumental activities of daily living (ADL/IADL) among PLWD; besides, about 1 in 5 PLWD received no care for their ADL/IADL limitations; and most importantly, the above absence of care has not improved over time in all studied countries, regardless of the stages of development. The patterns were evident for both ADL and IADL limitations and for informal and formal care. Our subgroup analysis showed consistency of these three facts, but also highlighted more worrying situations that about 2 in 5 PLWD received no care for their ADL limitations and at least 3 in 5 PLWD received no formal care. We also reported the greater absence of care for more vulnerable populations with less education and living alone. Our estimates highlight the urgency to ensure basic provision of care for PLWD with functional limitations, especially among those who are less educated or live alone.

